# Genome-wide association study reveals the genetic basis of brace root angle and diameter in maize

**DOI:** 10.3389/fgene.2022.963852

**Published:** 2022-10-06

**Authors:** Daqiu Sun, Sibo Chen, Zhenhai Cui, Jingwei Lin, Meiling Liu, Yueting Jin, Ao Zhang, Yuan Gao, Huiying Cao, Yanye Ruan

**Affiliations:** ^1^ Shenyang Key Laboratory of Maize Genomic Selection Breeding, Liaoning Province Research Center of Plant Genetic Engineering Technology, College of Biological Science and Technology, Shenyang Agricultural University, Shenyang, China; ^2^ Key Laboratory of Northern Geng Super Rice Breeding, Ministry of Education, Rice Research Institute, Shenyang Agricultural University, Shenyang, China; ^3^ Key Laboratory of Soybean Molecular Design Breeding, Northeast Institute of Geography and Agroecology, Chinese Academy of Sciences, Changchun, China

**Keywords:** bracing root, NAC transcription factor, cell wall, genetic architecture, SNP

## Abstract

Brace roots are the main organ to support the above-ground part of maize plant. It involves in plant growth and development by water absorption and lodging resistance. The bracing root angle (BRA) and diameter (BRD) are important components of brace root traits. Illuminating the genetic basis of BRA and BRD will contribute the improvement for mechanized harvest and increasing production. A GWAS of BRA and BRD was conducted using an associated panel composed of 508 inbred lines of maize. The broad-sense heritability of BRA and BRD was estimated to be respectively 71% ± 0.19 and 52% ± 0.14. The phenotypic variation of BRA and BRD in the non-stiff stalk subgroup (NSS) and the stiff stalk subgroup (SS) subgroups are significantly higher than that in the tropical/subtropical subgroup (TST) subgroups. In addition, BRA and BRD are significantly positive with plant height (PH), ear length (EL), and kernel number per row (KNPR). GWAS revealed 27 candidate genes within the threshold of *p* < 1.84 × 10^−6^ by both MLM and BLINK models. Among them, three genes, *GRMZM2G174736*, *GRMZM2G445169* and *GRMZM2G479243* were involved in cell wall function, and *GRMZM2G038073* encoded the NAC transcription factor family proteins. These results provide theoretical support for clarifying the genetic basis of brace roots traits.

## Introduction

Plant growth and development need root system to take up water and nutrients necessary to live (Su et al., 2020). In addition, the root system of maize (*Zea mays* L.) protects the plant from the wind and resist the lodging. The root system is composed of embryogenic and postembryonic roots in maize. Embryogenic roots include primary root and seminal roots, and post-embryonic roots include lateral roots and shoot-borne roots. Shoot-borne roots that form below the ground are called crown roots while those above the ground are called brace roots (Hochholdinger, 2009; Guo et al., 2018; Hochholdinger et al., 2018). Through morphological, anatomical and physiological approaches, considerable progress of the brace-root-related traits had been made, including lodging-resistance (Liu et al., 2012; Erndwein et al., 2020; Reneau et al., 2020) and nutrient and water acquisition (Wang et al., 2006; Van Deynze et al., 2018).

From a genetic perspective, maize brace root is a complex trait, which is governed by multiple quantitative trait loci (QTL). Over the last 30 years, QTL mapping has become a classic method for highlighting the genetic basis of continuous variation in a variety of systems. The QTLs for total brace root tier number (TBRTN) and effective brace root tier number (EBRTN) have been identified using recombinant inbred lines (RILs) and immortalized F2 (IF2) populations (Ku et al., 2012). Later, six brace root traits were detected in a F2 population (Gu et al., 2017). In addition, eight QTLs were identified for tier number (TN), root number (RN), and radius of the brace root (RBR) (Zhang et al., 2018a). Recently, twenty-one QTLs were identified in backcross population for 7 brace-root-related traits in maize (Sun et al., 2020).

Although genetic linkage analysis for QTL mapping is an effective tool, constructing segregating populations is necessary (Yu and Buckler, 2006). With the reducing cost and increasing throughput, next-generation sequencing (NGS) technologies have provided us with new opportunities to construct high-density genetic maps from genome-wide single nucleotide polymorphism (SNP) markers (Wang et al., 2019a; Benjamin et al., 2019; Song et al., 2020).

Genome-wide association analysis (GWAS) employed genotype and phenotype data of natural populations with extensive natural variation to find correlations between SNPs and a phenotype (Fong et al., 2010). It is an effective method to revealing the genetic basis of complex quantitative traits (Cui et al., 2016, 2018; Tao et al., 2020; Zhang et al., 2022; Zhao et al., 2022). At present, a total of 34 QTLs have been detected for 13 morphology traits of maize root, and single QTL explained 5.7%–15.9% of the phenotypic variance (Wang et al., 2019b). In addition, seven seedling root architectural traits were examined by integrating GWAS and QTL mapping (Moussa et al., 2021). QTLs involving the number node number of brace root, brace root number, and brace root dry weight were defined as relative phenotypic values of seedling traits under waterlogging conditions and were used to evaluate waterlogging tolerance in tropical maize (Guo et al., 2021). Liu et al. (2022) revealed a total of 9 SNPs that were significantly associated with metaxylem vessels in maize brace roots. The brace root angle (BRA) and brace root diameter (BRD) are also important traits of maize root and regulate by multiple genes, but the genetic basis is not yet clear. In this study, a GWAS analysis of 508 maize inbred lines with 543,641 SNPs genotypes was performed in three environments to analyze the phenotypic diversity and genetic basis of the brace root traits. The study also identified a range of candidate genes associated with BRA and BRD, providing a useful resource for further functional studies.

## Materials and methods

### Panel for association mapping

The GWAS association panel was comprised of 508 different maize inbred lines, including 60 from the United States’ Germplasm Enhancement of Maize, 223 from Mexico’s International Center for Maize Improvement (CIMMYT), and 225 from China’s germplasm resources. All resources were preserved by College of Biological Science and Technology of Shenyang Agricultural University. The majority of CIMMYT’s inbred lines came from tropical or subtropical regions, whereas most lines from the US and China came from temperate locations. Previous studies of the kinship of 508 maize inbred lines were based on K (model-based subgroups), and the maize panel was clustered into three clear subpopulations with 27 the stiff stalk (SS) inbred lines; 70 the non-stiff stalk (NSS) inbred lines; 196 the tropical-subtropical (TST) inbred lines, and the remaining 215 were classified into an admixed (MIXED) line. A previous study gave detailed information on the 508 inbred lines, including linkage disequilibrium, genetic diversity, and population structure (Yang et al., 2011; Jiang et al., 2020).

### Field experiments and phenotypic data collection

The association panel’s 508 inbred lines were planted in three locations in China: Shenyang City, Liaoning Province (LN) (123°25E, 41°48N) in 2016 and 2017, and Sniping, Jilin Province (JL) (123°17E, 42°31N) in Northeast China in 2016. All of the lines were planted using a randomized complete block design with two replicates. Each row is planted in rows 2-m long and 0.6-m wide, with a 0.4 m aisle in the middle of each plot.

**FIGURE 1:**
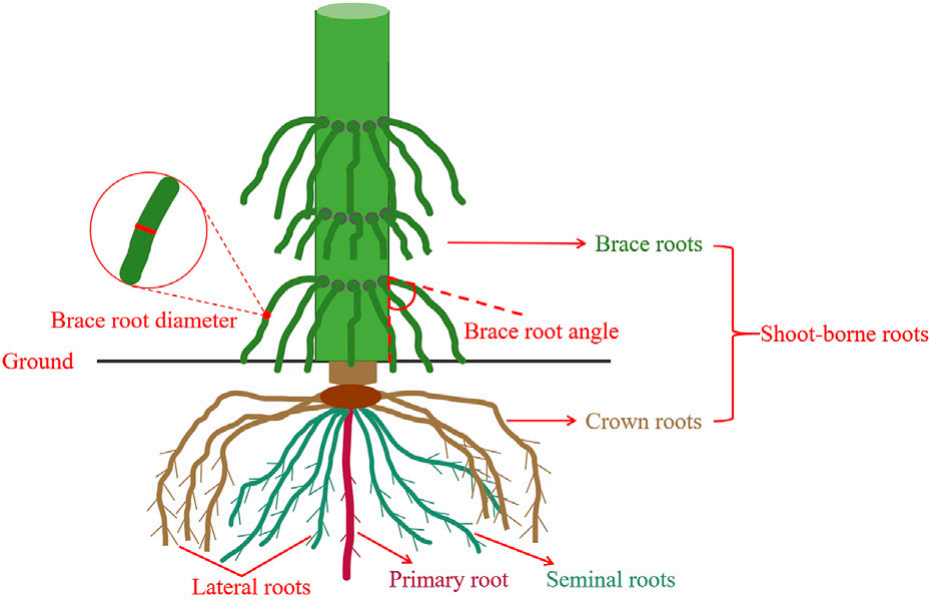
Measurement location of BRA and BRD in the field.

Maize brace roots traits were measured during reproductive growth. Brace roots in good condition, without cracking or wilting, were chosen to facilitate subsequent correlation analysis in order to obtain more accurate data. In each row, two the outermost brace roots close to the ground with mid-growing were selected for measurement. Measure the BRD using an electronic vernier caliper in the middle of the base of the brace roots (Centimeter). BRA was measured between the point of occurrence of the brace root and the main stem (degree) (Figure 1).

**FIGURE 2:**
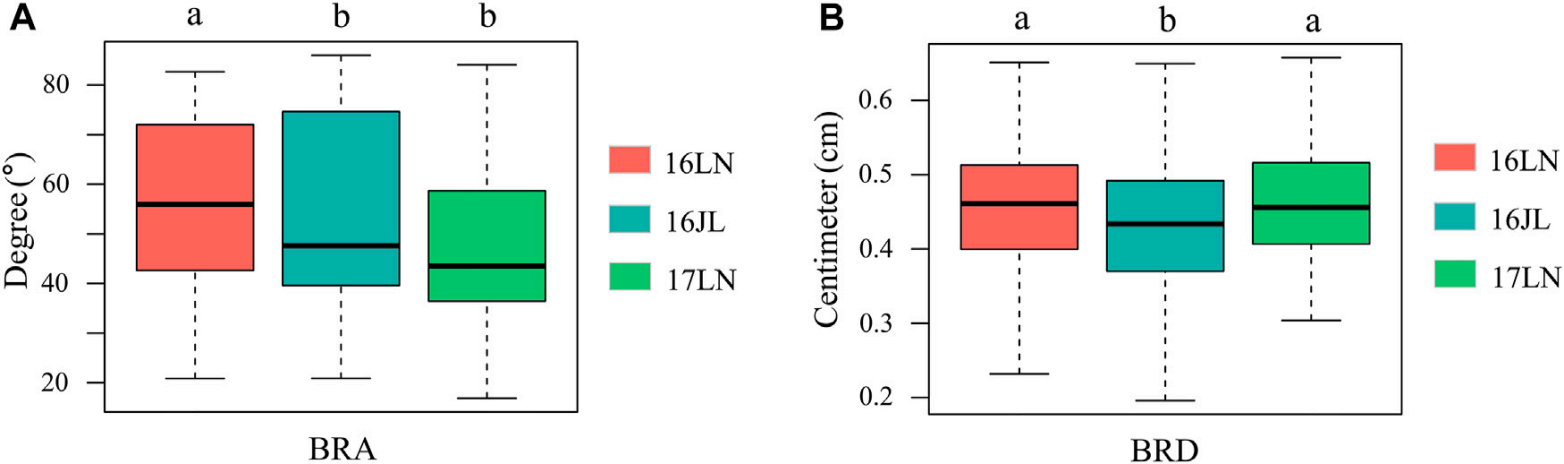
Boxplot of BRA and BRD of the three environments. **(A)** BRA, **(B)** BRD. ANOVA was used to examine phenotypic differences between different environments. a and b indicate statically significant differences at *p* ≤ 0.05. *16LN*, Liaoning Province in 2016; *16JL*, Jilin Province in 2016; *17LN*, Liaoning Province in 2017.

### Phenotype statistical analysis

A mixed linear model was used to calculate the best unbiased linear predictive value (BLUP) for the maize brace root traits, and the average value was added to the estimate to obtain the final BLUP value: $y_{ijk}=\mu +e_{l}+r_{k\left(l\right)}+f_{i}+{\left(fe\right)}_{il}+\varepsilon_{lik}$ , where *μ* represents the grand mean of brace root traits, and $f_{i}$ is the genetic effect of the *i*th line, $e_{l}$ represents environmental effect of the *l*th environment, ${\left(fe\right)}_{il}$ is the interaction effect between genetic and environmental effects, $r_{k\left(l\right)}$ is the repeated influence within the environment, and $\varepsilon_{lik}$ is the residual error. The “PROC MIXED” program in SAS software (Release 9.1.3; SAS Institute, Cary, NC, United States) was used to analyze the phenotypic variation of maize brace root traits. Broad-sense heritability is, respectively, calculated at individual environment and multiple environments as follows: $h^{2}=\frac{\sigma_{g}^{2}}{\sigma_{g}^{2}+\sigma_{e}^{2}/nreps}$ and $h^{2}=\frac{\sigma_{g}^{2}}{\sigma_{g}^{2}+\sigma_{ge}^{2}/nEnvs+\sigma_{e}^{2}/n\left(Envs\times reps\right)}$ , where $\sigma_{g}^{2}$ represents genetic variance, $\sigma_{e}^{2}$ represents a residual error, $\sigma_{ge}^{2}$ represents the interaction of genotype and environment, and *n* represents the number of environments and replications.

### Genome-wide association study

For the GWAS, the genotyping dataset was a whole genetic map assembled from the 50 K SNP array and RNA-seq, which comprised 543,641 SNP markers (minor allele frequency > 5%) (Maloof, 2007). The BLUP values of BRA and BRD in three individual environments and all environments were used to perform association analysis. For this study two association models were implemented namely Mixed Linear Model (MLM) and Bayesian information criterion and Linkage-disequilibrium Iteratively Nested Keyway (BLINK). K and Q matrices were considered in MLM using TASSEL V5.0 software package to avoid spurious associations (Bradbury et al., 2007). We then used the uniform Bonferroni-corrected threshold at *α* = 1 for MLM reported in previous studies as a significance cutoff (Li et al., 2013; Yang et al., 2014; Mao et al., 2015). BLINK model eliminates the assumption to improve statistical power by using the linkage disequilibrium (LD) method. Markers are sorted with the most significantly associated maker on the top as reference. The remaining markers are removed if they are in LD with the most associated marker. Among the remaining makers, the most significantly associated maker is selected as the reference. The process is repeated until no markers can be removed. *p*-value was the probability of observing at least the same sample as the actual observed sample when the null hypothesis was true in the hypothesis test. The *p*-value for this study was calculated by 1/n (*n* = 543,641) with a *p*-value of 1.84 × 10^−6^ as the final significance cutoff in the association analysis. Use the ANOVA function in the R package to estimate the contribution of the SNPs to the phenotypic variance. After adjusting for the population structure effects, the R^2^ of each significant SNP was calculated using two linear models: $Y=X_{i}\alpha_{i}+P\beta +\varepsilon$ , which was used to estimate the total variance of all significant SNPs, and $Y=X_{\alpha }+P\beta +\varepsilon$ , which was used to estimate the variance of individual significant SNPs.

In these models, Y and X represent phenotype and SNP genotype vectors, respectively, P is the matrix of the three subgroups (NSS, SS, TST), and *α*, *β* and *ε* are SNP, subgroup and random residual random effects, respectively. *α* and *β* are unknown vector containing fixed effects, and ε vectors are random effects assumed to be normally distributed.

### Annotation of candidate genes

Find the physical locations of the SNPs in the B73 RefGen_v2 genome (www.maizesequence.org). Identify genes within 50-kb (R^2^ < 0.2) upstream and downstream of significantly associated SNPs loci (Cui et al., 2020; Jiang et al., 2020) and functionally annotate homologous genes in rice and *Arabidopsis thaliana*.

### Expression heat-map of candidate genes

Download the expression levels of candidate genes in different tissues of maize B73 from the MaizeGDB qTeller database (https://qteller.maizegdb.org/). The value used to make the heat-map was log_10_ (n + 1), where n represents the TPM value.

## Results

### Brace root diversity and heritability

The phenotypic data and the BLUP values of 508 maize inbred lines were shown in Supplementary Table S1. BRA and BRD exhibited slightly left-skewed normal distributions (Supplementary Figure S1). In addition, BRA and BRD extensive phenotypic variation in different environments in the association panel (Figure 2). The distribution range of BRA was from 19.24° to 84.15°, and BRD was from 0.25 to 0.65 cm (Supplementary Figure S1). Variance analysis indicated that genotype variance had a significant effect (*p* < 0.01) in a single environment and across all environments. Furthermore, environment variance and genotype × environment (G × E) interaction variance were highly significant (*p* < 0.01) across environments. The broad-sense heritability was 71% ± 0.19 and 52% ± 0.14, respectively, indicating that the phenotypic variation of BRA and BRD was mainly derived from genetic factors (Table 1; Supplementary Table S2).

**TABLE 1:** Phenotypic variation distribution, analysis of variance, and broad-sense heritability of BRA and BRD.

Traits^a^	Means ± SD	Range	Variance component^b^ ^,^ ^c^			*h* ^2^ ± SD^d^
			Genotype (G)	Environment (E)	G × E	
BRA	52.93 ± 8.10	19.24–84.15	37.59*	10.96*	26.46*	0.71 ± 0.19
BRD	0.46 ± 0.05	0.25–0.65	29.15*	16.38*	13.56*	0.52 ± 0.14

a *Traits*, the best linear unbiased prediction; *BRA*, brace root angle; *BRD*, brace root diameter.

b *G* and *E* indicate genotype and environment, respectively, and *G × E* indicate interaction of G and E.

c *represents significant differences at the 0.01 level.

d Family mean-based broad-sense heritability.

**FIGURE 3:**
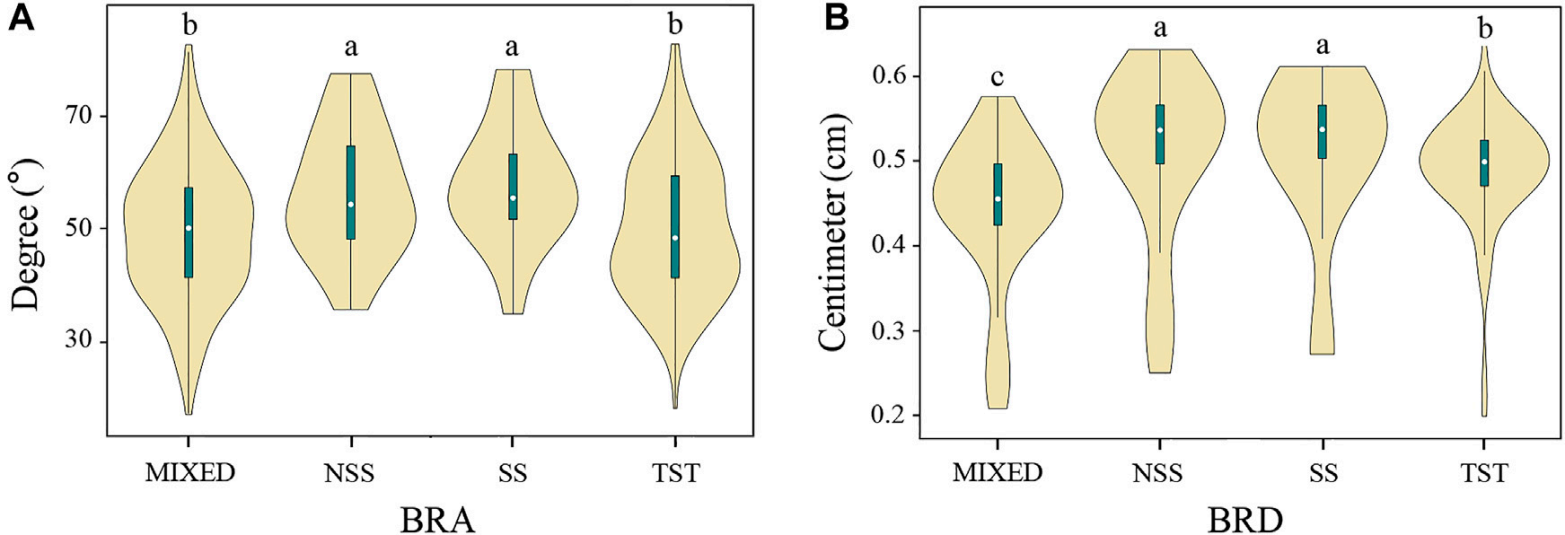
Violin plot of BRA and BRD of the four major subgroups. **(A)** BRA, **(B)** BRD. *SS*, the stiff stalk subgroup; *NSS*, the non-stiff stalk subgroup; *TST*, the tropical-subtropical subgroup; *MIXED*, admixed subgroups. ANOVA was used to examine phenotypic differences between different subgroups. a, b and c indicate statically significant differences at *p* ≤ 0.05.

The association panel used in this study can be divided into four subpopulations: SS, NSS, TST, and MIXED (Yang et al., 2011). The SS and NSS subpopulations are from the temperate zone, the TST subpopulation is from the tropics or subtropics, and the MIXED subpopulation contains the remaining non-classified inbred lines (Yang et al., 2011). Therefore, the phenotypic variation of BRA and BRD between different subgroups was compared to investigate the effect of population structure (Figure 3). Among them, the medians of BRA and BRD in NSS and SS subgroups were significantly higher than that in other subgroups, and the BRA phenotype variation range in NSS and SS subgroups was smaller than that in other subgroups. In summary, the BRA and BRD traits exhibit wide variation according to genetic backgrounds, population structure and environment, and were suitable for GWAS analysis.

### Correlations of bracing root angle and bracing root diameter phenotypes with other plant developmental processes

**FIGURE 4:**
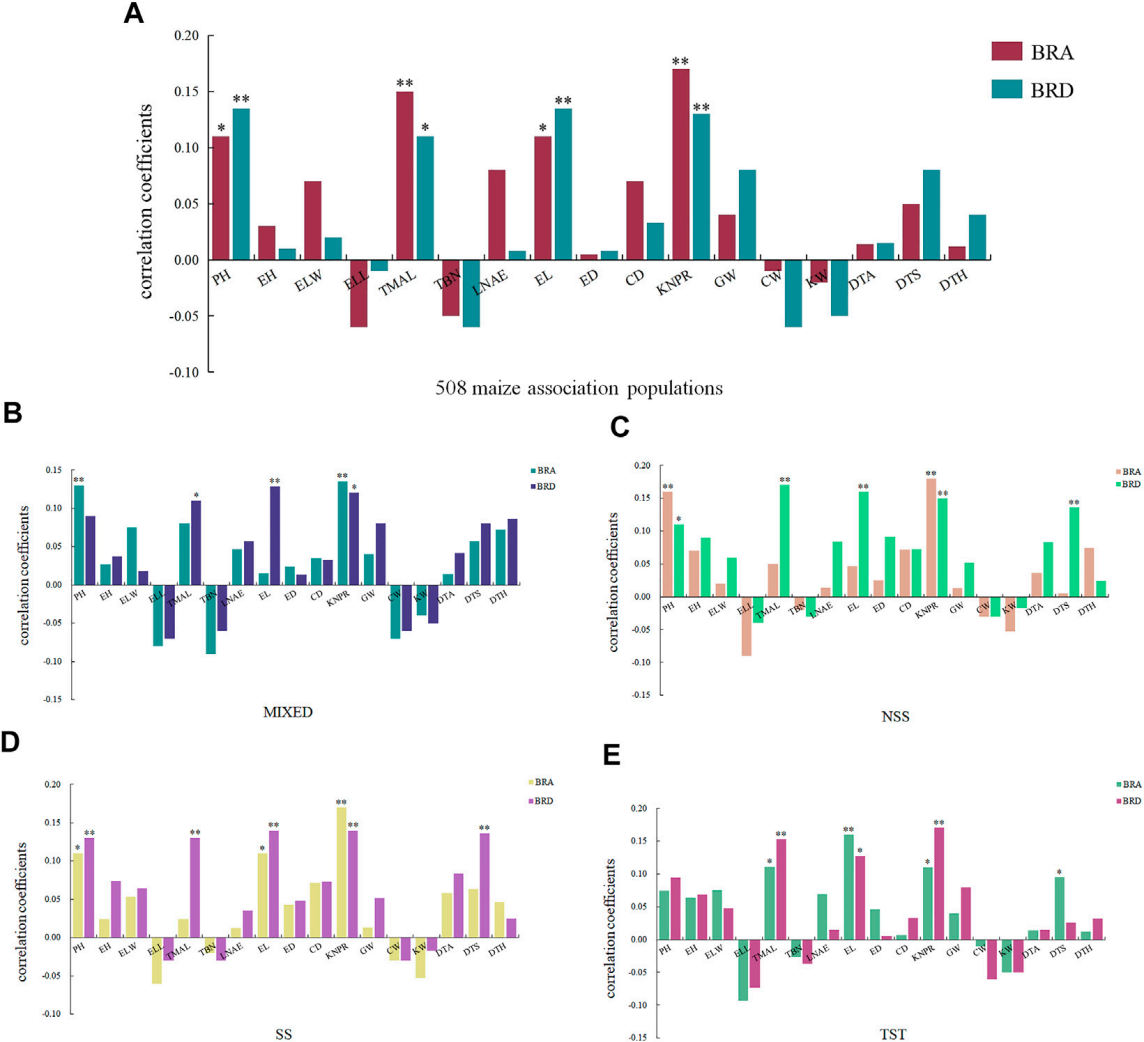
Correlation between BRA, BRD and 17 agronomic traits. **(A)** 508 maize association populations, **(B)** MIXED, **(C)** NSS, **(D)** SS, **(E)** TST. *BRA*, brace root angle; *BRD*, brace root diameter; *PH*, plant height; *EH*, ear height; *ELW*, ear leaf width; *ELL*, ear leaf length; *TMAL*, tassel maximum axis length; *TBN*, tassel branch number; *LNAE*, leaf number above ear; *EL*, ear length; *ED*, ear diameter; *CD*,cob diameter; *KNPR*, kernel number per row; *GW*, 100-grain weight; *CW*, cob weight; *KW*, kernel width; *DTA*, days to anthesis; *DTS*, days to silking; *DTH*, days to heading. * represents a significant differences at the 0.05 level; *** represents significant differences at the 0.01 level.

As an integral part of the root system, brace roots might be related to the growth and development of other agronomic traits. To further clarify the relationships between BRA and BRD with other agronomic traits, we performed a correlation analysis. Correlation studies were conducted on 17 reported agronomic traits using the same 508 maize inbred lines (Yang et al., 2014). Based on Pearson’s correlation coefficients, a positive correlation exists between BRA and tassel maximum axis length and kernel number per row at 1% significant level, and plant height and ear length at 5% significant level. A positive correlation exists between BRD and plant height, ear length and kernel number per row at 1% significant level, and tassel maximum axis length at 5% significant level. These results indicated that BRA and BRD can affect plant morphology and yield (Figure 4).

The 508 maize inbred lines was clustered into three subpopulation and an admixed lines (Yang et al., 2014). We revealed the effect of population structure on 17 agronomic traits. As shown in Figure 4A, a significant correlation existed between BRD and tassel maximum axis length, ear length in all four subpopulations. There were 3,5,5,3 traits existing significant correlation with BRD in MIXED, NSS, SS and TST subpopulation respectively (Figures 4B–E). In comparison, there were only 2,2,3,4 traits with BRA, indicating population structure had an important impact on agronomic traits, and BRD was more closely related to yield.

**FIGURE 5:**
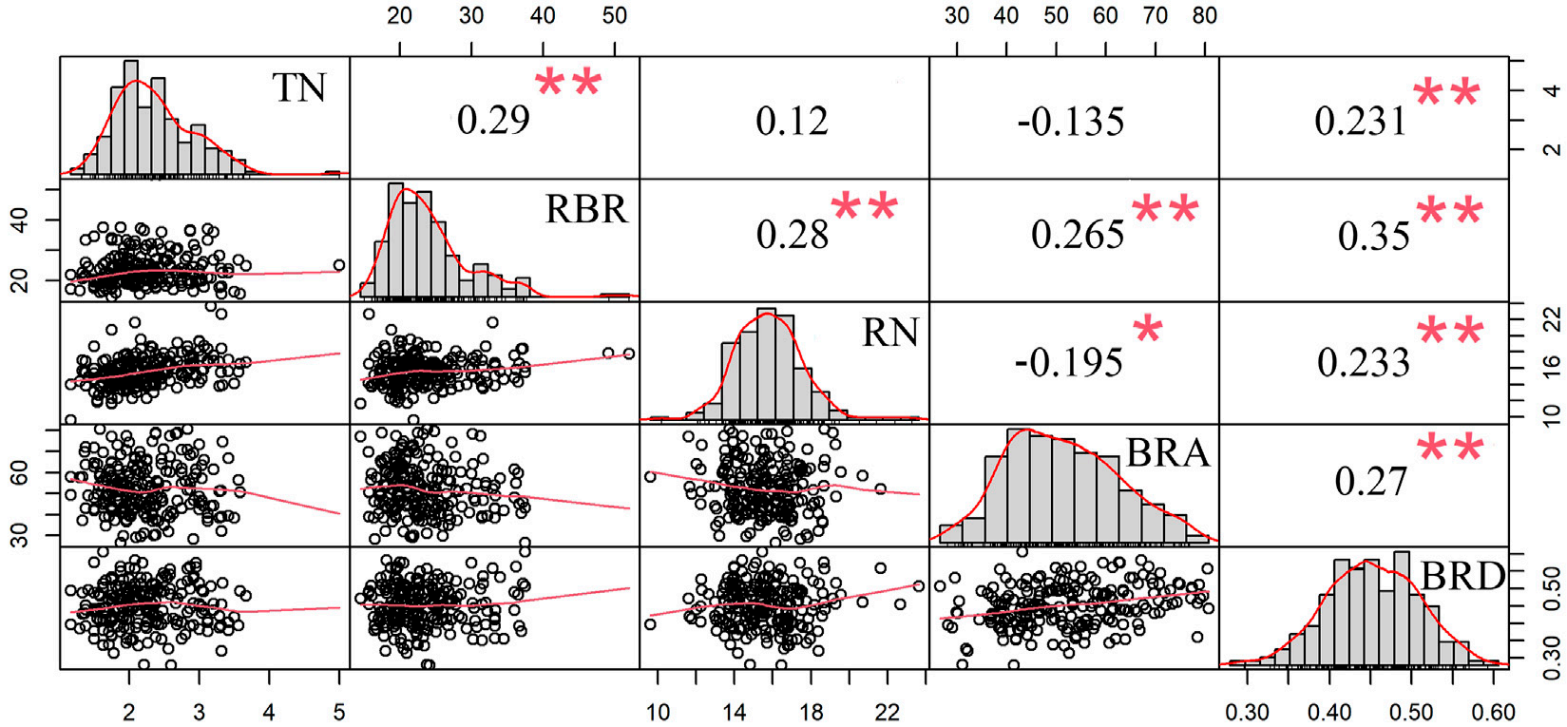
Correlations between BRA, BRD and three other brace root traits. The diagonal plot represents the frequency of the phenotypic distribution between the brace root angle, the brace root diameter, and the other three brace root traits. The value above the diagonal is Pearson’s correlation coefficient between the two traits. Below the diagonal is a scatter plot of two traits. * represents significant difference at the 0.05 level; ** represents significant differences at the 0.01 level. *BRA*, brace root angle, *BRD*, brace root diameter, *TN*, tier number of the brace root, *RBR*, radius of the brace root, *RN*, number of the brace root.

In addition, the correlations between BRA, BRD and brace root tier number (TN), radius of the brace root (RBR, r = C/2π, where C is the outer circumference of the circle described by brace roots striking into the soil) and brace root number (RN) (Zhang et al., 2018a) were also analyzed. As shown in Figure 5, all five brace root traits also followed normal distributions. BRA was positively correlated with RBR (r = 0.265, *p* < 0.01), whereas was negatively correlated with RN (r = −0.195, *p* < 0.05). BRD was positively correlated with RBR (r = 0.35, *p* < 0.01), TN (r = 0.231, *p* < 0.01) and RN (r = 0.233, *p* < 0.01). Furthermore, BRA was positively correlated with BRD (r = 0.27, *p* < 0.01).

### Genome-wide association analysis

**FIGURE 6:**
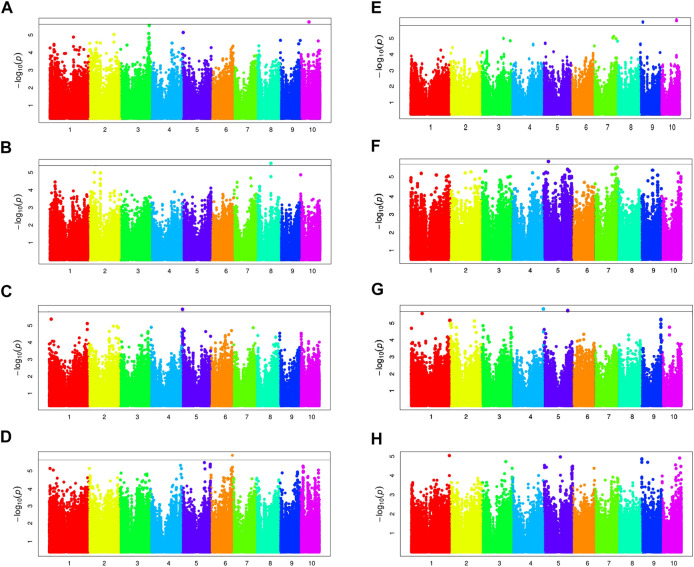
Manhattan plots of BRA and BRD by MLM. Manhattan plots for *BRA-BLUP*, *16LN-BRA*, *16JL-BRA*, *17LN-BRA*, *BRD-BLUP*, *16LN-BRD*, *16JL-BRD* and *17LN-BRD* are shown in **(A)**, **(B)**, **(C)**, **(D)**, **(E)**, **(F)**, **(G)** and **(H)**, respectively. The black lines show genome-wide significance at stringent thresholds of 1.84 × 10^−6^.

To reduce the impact of environmental change, phenotypic BLUP values in all environments and three individual environments (16LN, 16JL, and 17LN) were used for association studies. The GWAS results of BRA and BRD with MLM and BLINK model are displayed in Figure 6; Table 2 (Supplementary Figures S2–S4). For BRA, we detected a total of six independently significant SNPs by two models. BLINK captured all six SNPs, but MLM did only five. For BRD, BLINK captured 9 significant SNPs, including all 5 SNPs identified by MLM. But no significant SNP was found by both methods for 17LN.

**TABLE 2:** Positions of SNPs significantly correlated with BRA and BRD were Co located by MLM and BLINK.

Traits	SNP	Chr	Positions (bp)	Allele^a^	*p*-value	R^2^ (%)^b^
BRA	chr3.S_218194187	3	218194187	A/G	4.91492E-07	2.40
	chr10.S_4090245	10	4090245	G/A	5.36755E-07	10.90
	chr8.S_107094508	8	107094508	T/C	2.99367E-07	3.50
	chr5.S_3504616	5	3504616	C/T	1.93558E-07	5.70
	chr6.S_112435160	6	112435160	A/C	5.45E-07	0.45
BRD	chr9.S_140361053	9	140361053	G/T	4.24628E-07	14.70
	chr10.S_95437751	10	95437751	A/C	1.71503E-06	13.95
	chr5.S_2275215	5	2275215	G/A	2.87E-07	2.40
	chr4.S_237033434	4	237033434	G/T	1.86264E-07	4.90
	chr5.S_180407216	5	180407216	G/C	2.29814E-07	8.35

a Major/minor alleles, underlined bases indicate favorable alleles.

b Percentage of phenotypic variation explained by the cumulative effect of a single significant SNP.

**FIGURE 7:**
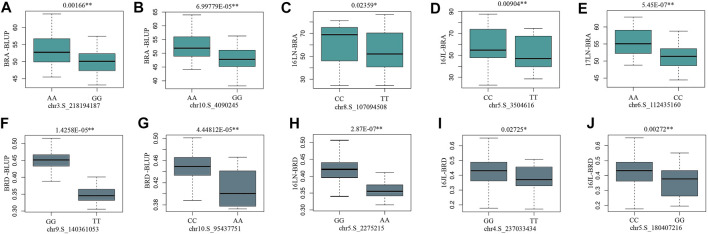
The boxplot of phenotypic differences between the major and minor alleles of significant SNPs associated with BRA and BRD. The *p*-values (Student’s *t*-test) of the allelic effects of BRA and BRD are exhibited above each small plot. **(A)** *BRA-BLUP*, **(B)** *BRA-BLUP*, **(C)** *16LN-BRA*, **(D)** *16JL-BRA*, **(E)** *17LN-BRA*, **(F)** *BRD-BLUP*, **(G)** *BRD-BLUP*, **(H)** *16LN-BRD*, **(I)** *16JL-BRD*, **(J)** *16JL-BRD*.

MLM and BLINK are two different statistical methods for GWAS. MLM includes the kinship matrix (K) as an additional random effect component (Kumar et al., 2022), whereas BLINK uses a multiple loci test method instead of a single loci test method, by combining a fixed effect model (FEM), Bayesian information criteria, and linkage disequilibrium information (Huang et al., 2017). Due to the less false-negative rate, more significant SNPs had been revealed by BLINK model in the present study. There are 10 significant SNPs identified by both BLINK and MLM, five for BRA and the rest for BRD. Among them, the allele effects of chr5.S_2275215 was the most significant for BRD phenotypic variation, with a *p*-value of 2.87E-07 (Figure 7). According to MLM method, the five SNPs of BRA explained 2.40%–10.90% of phenotypic variation; and the five SNPs of BRD explaining 13.95%–14.70% of phenotypic variation (Table 2; Supplementary Table S3).

### Expression pattern of candidate gene in different maize tissues

**FIGURE 8:**
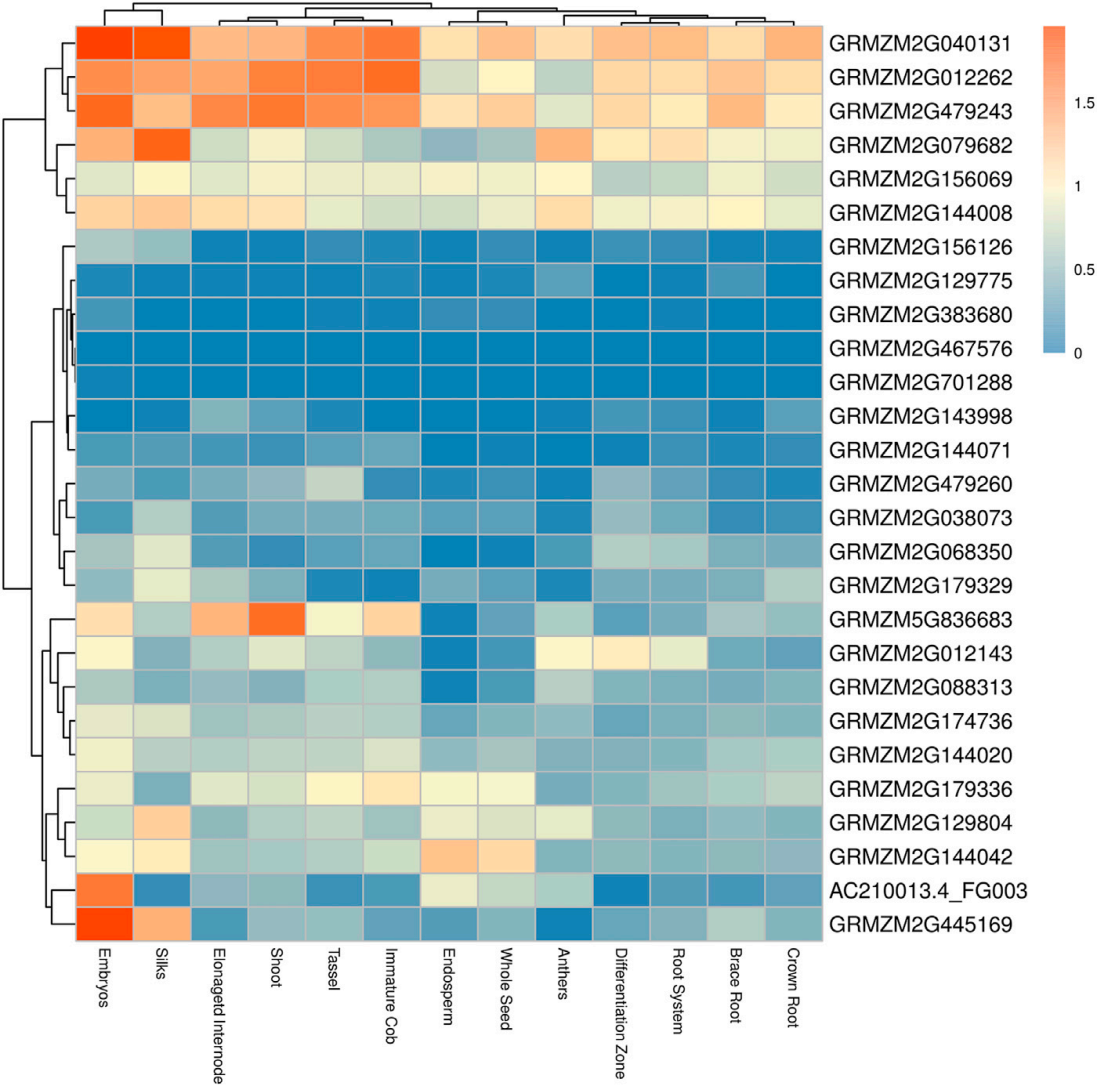
Expression patterns of candidate genes identified by MLM. Each expression in the graph is the log_10_ (n + 1) transformed value of TPM counts for brace root and other tissues, as indicated at the bottom of each column. Rows and columns are distributed according to similarity (cluster analysis on top and left). Orange, white and blue represent higher, moderate and lower genes expressed in each tissue, respectively.

Ten SNPs that were significantly associated with BRA and BRD were revealed by both MLM and BLINK. A total of 27 candidate genes were identified within 50-kb upstream and downstream of each SNP, of which 25 genes were functionally annotated. According to the functional annotation, the candidate gene *GRMZM2G038073* identified by 16-JL-BRD encoded the NAC (No Apical Meristem) domain transcriptional regulator superfamily protein. In addition, three candidate genes *GRMZM2G479243*, *GRMZM2G174736* and *GRMZM2G445169* identified by BRD and 16-LN-BRD are involved in cell wall functions (Supplementary Table S3). To further determine the expression levels of candidate genes within the loci-linked interval of significant SNPs in each tissue, the expression patterns of published RNA-seq datasets from 13 different organs/tissues, including brace root, were analyzed (Figure 8). There was no tissue-specific expression in any 27 genes. The gene *GRMZM2G040131* encoding methyl-binding protein had relatively high expression levels in various tissues. The three genes involved in cell wall functions showed expression from moderate to high level. But the gene *GRMZM2G038073* encoded the NAC transcriptional factor had a lower expression.

## Discussion

### Genetic basis of bracing root angle and bracing root diameter

Since maize is one of the most important crops for global food security, several efforts have been undertaken addressing the efficient utilization of germplasm collections for breeding purposes (Maldonado et al., 2019). In the present study, a genetically diverse association panel consisting of different maize inbred lines, were used for GWAS analysis of root traits of importance in the maize crop. BRD and BRA exhibited wide variation according to genetic backgrounds, and were moderately to highly heritable traits. Genetic and environmental effects are significant as is the interaction of genetic and environmental effects for BRA and BRD traits and is suitable for further GWAS analysis. Furthermore, the phenotypic variation among different environments is consistent with the variance analysis results, indicating that different environments can influence the phenotypic changes of BRA and BRD. Thus, the improvement of maize BRA and BRD can be through breeding programs in specific environments binding to the identified environment-specific significant SNPs.

GWAS is straightforward for data generation, management, and analysis (Purcell et al., 2007; Xiao et al., 2007; Pruim et al., 2010; Chang et al., 2015). It can also discover new biological mechanisms using reliable genotyping techniques (Hirschhorn, 2009; Visscher et al., 2017). However, it also has certain limitations. For example, all genetic determinants of complex traits cannot be fully identified by GWAS (Altshuler et al., 2008), and heritability for complex traits is difficult to accurately estimate (Visscher et al., 2008; Zuk et al., 2012). In addition, GWAS does not necessarily pinpoint causal variants and target genes (Altshuler et al., 2008), and often requires additional steps for identification, such as the development of new methods and fine-mapping (Mägi et al., 2017; Ng et al., 2017). Furthermore, population stratification is a difficult problem in genetic association studies that, if not considered, can lead to spurious associations (McClellan and King, 2010).

Maize originated and domesticated in the tropics and was subsequently grown and improved in subtropical and temperate regions. Therefore, population structure may have imposed effects on maize morphology due to different kinship of inbred lines in associated populations (Camus-Kulandaivelu et al., 2006). By comparing the phenotypic variation of BRA and BRD in different maize subpopulations, it was found that temperate regions have wider BRA and thicker BRD than tropical regions (Figure 3). It has been reported that rapid water evaporative in the tropics makes brace roots susceptible to drought stress, resulting in decreased expression of the *ZmRHCP1* gene, thereby reducing brace root yield (Vadez et al., 2012; Li et al., 2017). Temperate maize may have more brace roots and fewer crown roots, which may improve root-lodging resistance and water and nitrogen uptake (Zhang et al., 2018b). Therefore, there was difference in BRA and BRD between subgroups that may be affected by consanguinity.

### Coordination of bracing root angle and bracing root diameter with other processes of plant development

Correlations among brace root traits have been reported in previous studies. Zhang et al. indicated that BRA was positively correlated with brace root deployment width and negatively correlated with the number of whorls (Zhang et al., 2018b). In addition, BRD was positively correlated with the number of the brace root and the tier number of the brace root (Liu et al., 2012; Gu et al., 2017). The above results are consistent with ours. All these traits can affect root lodging resistance.

Plant height, reduced during the Green Revolution, has been associated with crop yield by affecting lodging tolerance in cereals. BRD is an important phenotype for resistance to root lodging. Our results showed that BRD was highly significantly positively correlated with plant height. However, Sharma and Carena (2016) reported that there was no correlation between plant height and root lodging during natural root lodging events. Notable, Hostetler et al. (2021) described the low positive correlations between brace root phenotypes and plant height, and analyzed their opposing effects on lodging susceptibility. The taller plants are susceptible to lodging, but stronger brace roots provide them with more lodging-resistance. These results indicate the complex relationship among plant height, brace root traits, and lodging-resistance. In addition, population structure is also an important factor affecting the correlation between different phenotypic traits.

### Candidate genes and pathways involved in brace root morphogenesis

The NAC family is a plant-specific transcription factor that plays an important role in plant development, various abiotic stress responses, and disease resistance (Yuan et al., 2019). *AtNAC2* (*ANAC092*) and *AtNAC1* (*ANAC021*) have been shown to promote the formation of lateral roots by gene overexpression in *Arabidopsis* (Xie, 2000; He et al., 2010). In monocot barley, there are three *HvNAC* genes upregulated highly in the root of three leaf stage, which belong to subfamily NAC-d, as do both *AtNAC2* and *AtNAC1*(Christiansen et al., 2011). In maize, there are 157 NAC family members divided into two large groups including 18 subgroups (Lu et al., 2015). In this study, we identified a candidate gene *GRMZM2G038073* (*Zm00001d017084*), which was annotated as NAC (No Apical Meristem) domain transcriptional regulator superfamily protein. Mao et al. (2015) identified a NAC gene (*ZmNAC111*) associated with natural variation in maize drought tolerance using GWAS. *ZmNAC111* overexpression in maize seedling improved drought tolerance and water-use efficiency. But *ZmNAC111* showed a lower expression in root due to an insertion of 82-bp miniature inverted-repeat transposable element (MITE) in the promoter. Interestingly, according to hot map of gene expression, *GRMZM2G038073* showed a lower expression in brace root, too. And then, we blasted the upstream sequences of *GRMZM2G038073* using the 82-bp MITE as a query and found a highly homologous DNA sequence located 3574-bp upstream of the start codon. Thus, we speculated that *GRMZM2G038073* may involve in root development and water absorption.

Plant cells are surrounded by a rigid wall, which provides mechanical protection, cellular stability, and cell-to-cell communication (Nicolas et al., 2001). The cell wall is composed of carbohydrates and structural proteins. *GRMZM2G174736*, a candidate gene of BRD, encodes the structural proteins hydroxyproline-rich-glycoproteins (HRGPs), which are also called extensions and take part in cell wall assembly (Saha et al., 2013; Chen et al., 2015; Borassi et al., 2021). It is known that the expression of HRGPs in a tissue-specific or development-specific manner (Keller and Lamb, 1989; Ye and Varner, 1990) and involve in root development (Ji et al., 1998, 1; Chen et al., 2015; Pinski et al., 2021). Opposite to extensions, expansins, encoded by *GRMZM2G445169*, are cell wall loosening protein to be responsible for organ growth in plants (Cosgrove, 2000; Choi et al., 2008; Pena et al., 2015). Kwasniewski and Szarejko (2006) demonstrated that the expansin EXPB1 was involved in root hair initiation, and Yu et al. (2011) reported that root hair-specific expansin EXPA17 was necessary in root hair elongation in rice. *GRMZM2G479243* encodes Arabidopsis FEI homologous-protein, which belongs to leucine-rich repeat (LRR) protein kinase family. The *fei1 fei2* double mutant in *Arabidopsis* caused a swollen-root phenotype, reduced cellulose production in roots and hypersensitivity to inhibition of cellulose biosynthesis (Xu et al., 2008). The cell walls are dynamic structures that respond to developmental and environmental changes (Debarati et al., 2016). All three genes, *GRMZM2G174736, GRMZM2G445169* and *GRMZM2G479243*, are supposed to take part in brace roots development *via* regulating cell wall function.

## Conclusion

In this study, we revealed the genetic basis of brace root diameter and brace root angle under the maize natural variant population, which exhibited wide variation according to genetic backgrounds, and were moderately to highly heritable, respectively. Their phenotypic variation was significantly higher in the non-stiff stalk subgroup and stiff stalk subgroups than in the tropical-subtropical subgroups. The brace root angle and brace root diameter existed significant correlation with different agronomic traits in different subgroups, and brace root diameter had more agronomic correlation, indicating population structure had an important impact on agronomic traits. The genome-wide association study revealed associations with 27 candidate genes. According to the published RNA-seq datasets from 13 different organs/tissues, there is no tissue-specific expression in any 27 genes. The gene *GRMZM2G040131* encoding methyl-binding protein had relatively high expression levels in various tissues. The three genes involved in cell wall functions showed expression from moderate to high level. But the gene *GRMZM2G038073* encoded the NAC transcriptional factor had a lower expression. This will provide a theoretical basis for the improvement of maize brace roots.

## Data Availability

Genotype and phenotype data used in this study have been uploaded to “https://datahold.cn/2022/09/29/brace-root-angle-and-diameter-of-association-population/”.

## Supplementary material

SUPPLEMENTARY FIGURE S1 Brace root angle and brace root diameter normal distribution analysis., **(A)**, BRA, **(B)**, BRD.

SUPPLEMENTARY FIGURE S2 Q-Q plots of BRA and BRD by MLM. Q-Q plots for BRA-BLUP, 16LN-BRA, 16JL-BRA, 17LN-BRA, BRD-BLUP, 16LN-BRD, 16JL-BRD and 17LN-BRD are shown in **(A)**, **(B)**, **(C)**, **(D)**, **(E)**, **(F)**, **(G)** and **(H)**, respectively.

SUPPLEMENTARY FIGURE S3 Manhattan plots of BRA and BRD by BLINK. Manhattan plots for BRA-BLUP, 16LN-BRA, 16JL-BRA, 17LN-BRA, BRD-BLUP, 16LN-BRD, 16JL-BRD and 17LN-BRD are shown in **(A)**, **(B)**, **(C)**, **(D)**, **(E)**, **(F)**, **(G)** and **(H)**, respectively. The black lines show genome-wide significance at stringent thresholds of 1.84 × 10^−6^.

SUPPLEMENTARY FIGURE S4 Q-Q plots of BRA and BRD by BLINK. Q-Q plots for BRA-BLUP, 16LN-BRA, 16JL-BRA, 17LN-BRA, BRD-BLUP, 16LN-BRD, 16JL-BRD and 17LN-BRD are shown in **(A)**, **(B)**, **(C)**, **(D)**, **(E)**, **(F)**, **(G)** and **(H)**, respectively.

## Abbreviations

BRA, brace root angle; BRD, brace root diameter; GWAS, genome-wide association study; BLUP, the best linear unbiased prediction; SNP, single nucleotide polymorphism; PH, plant height; EH, ear height; ELW, ear leaf width; ELL, ear leaf length; TMAL, tassel maximum axis length; TBN, tassel branch number; LNAE, leaf number above ear; EL, ear length; ED, ear diameter; CD, cob diameter; KNPR, kernel number per row; GW, 100-grain weight; CW, cob weight; KW, kernel width; DTA, days to anthesis; DTS, days to silking; DTH, days to heading; TN, tier number of the brace root; RBR, radius of the brace root; RN, number of the brace root; LN, Liaoning; JL, Jilin.
