# Supplementary figures and images for: Genome-wide association study reveals the genetic basis of brace root angle and diameter in maize
Source: Front Genet. 2022 Oct 6;13:963852. doi: 10.3389/fgene.2022.963852 (PMC9582141; doi:10.3389/fgene.2022.963852)

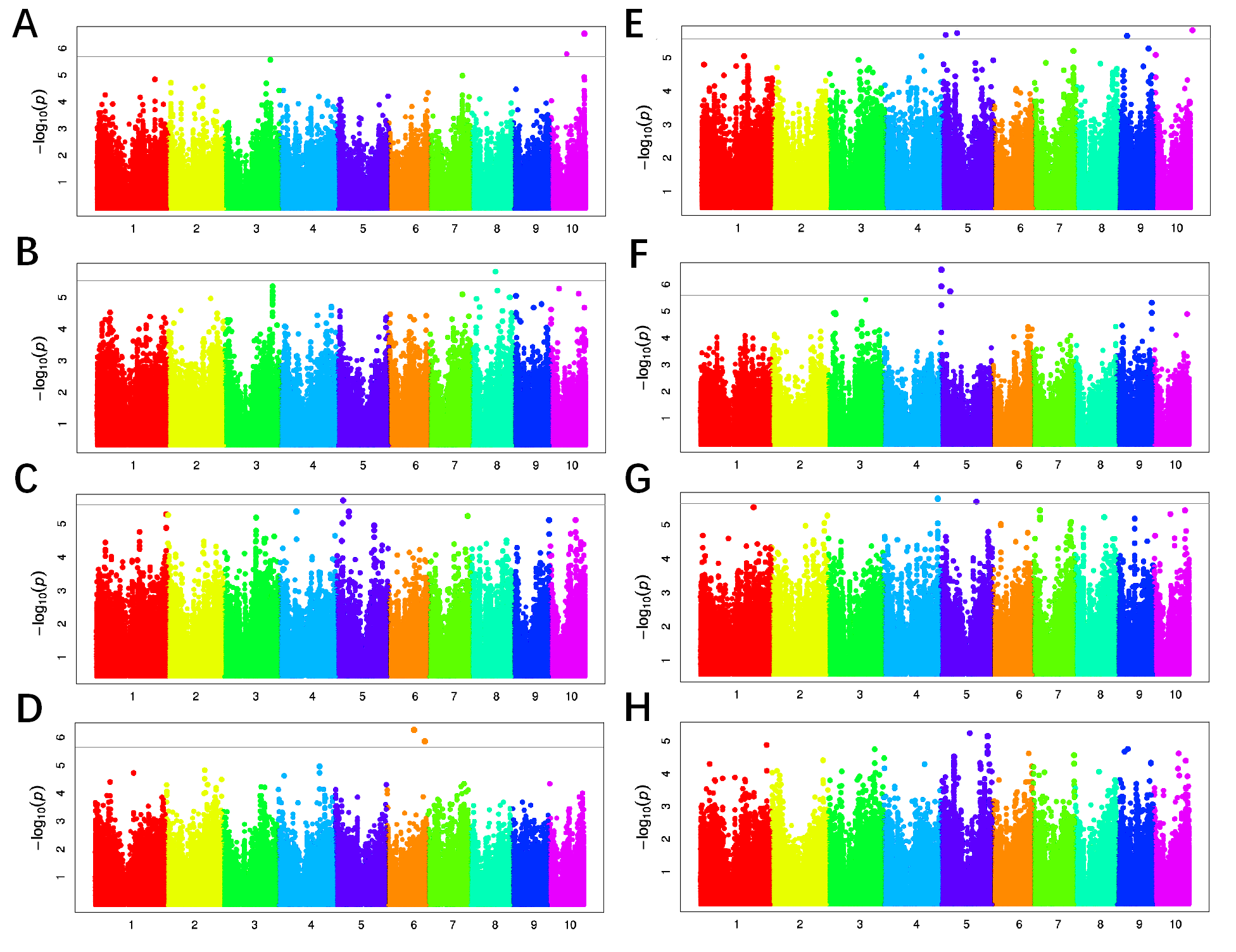

Supplement: Supplementary file 1 [file Image3.JPEG]

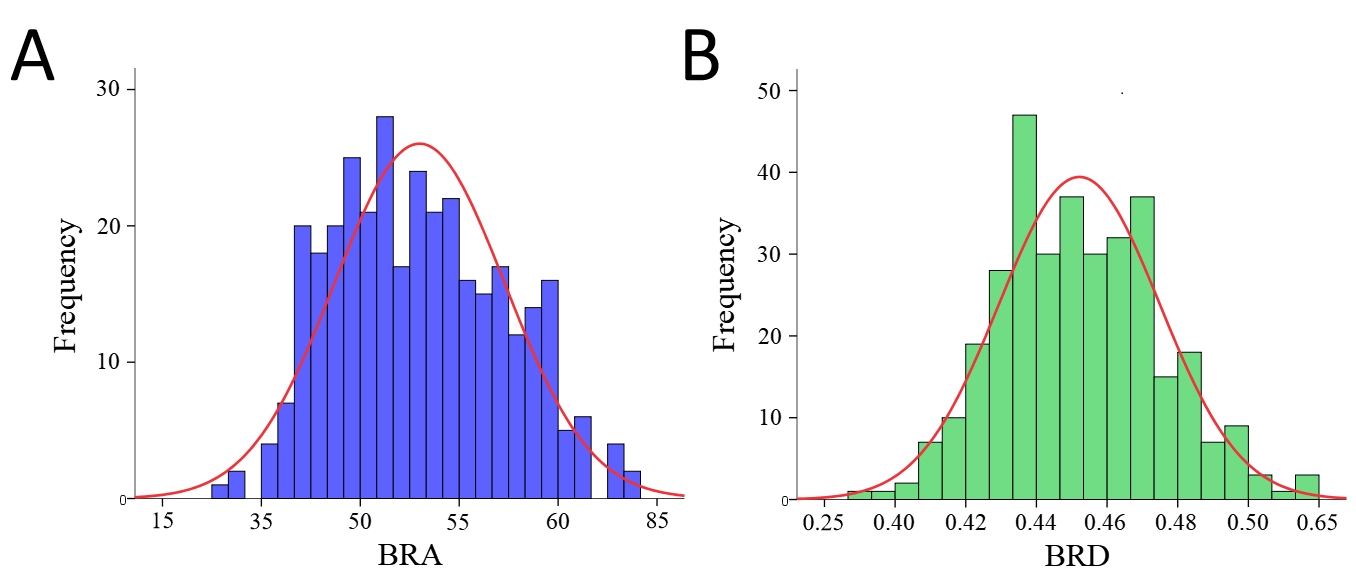

Supplement: Supplementary file 3 [file Image1.JPEG]

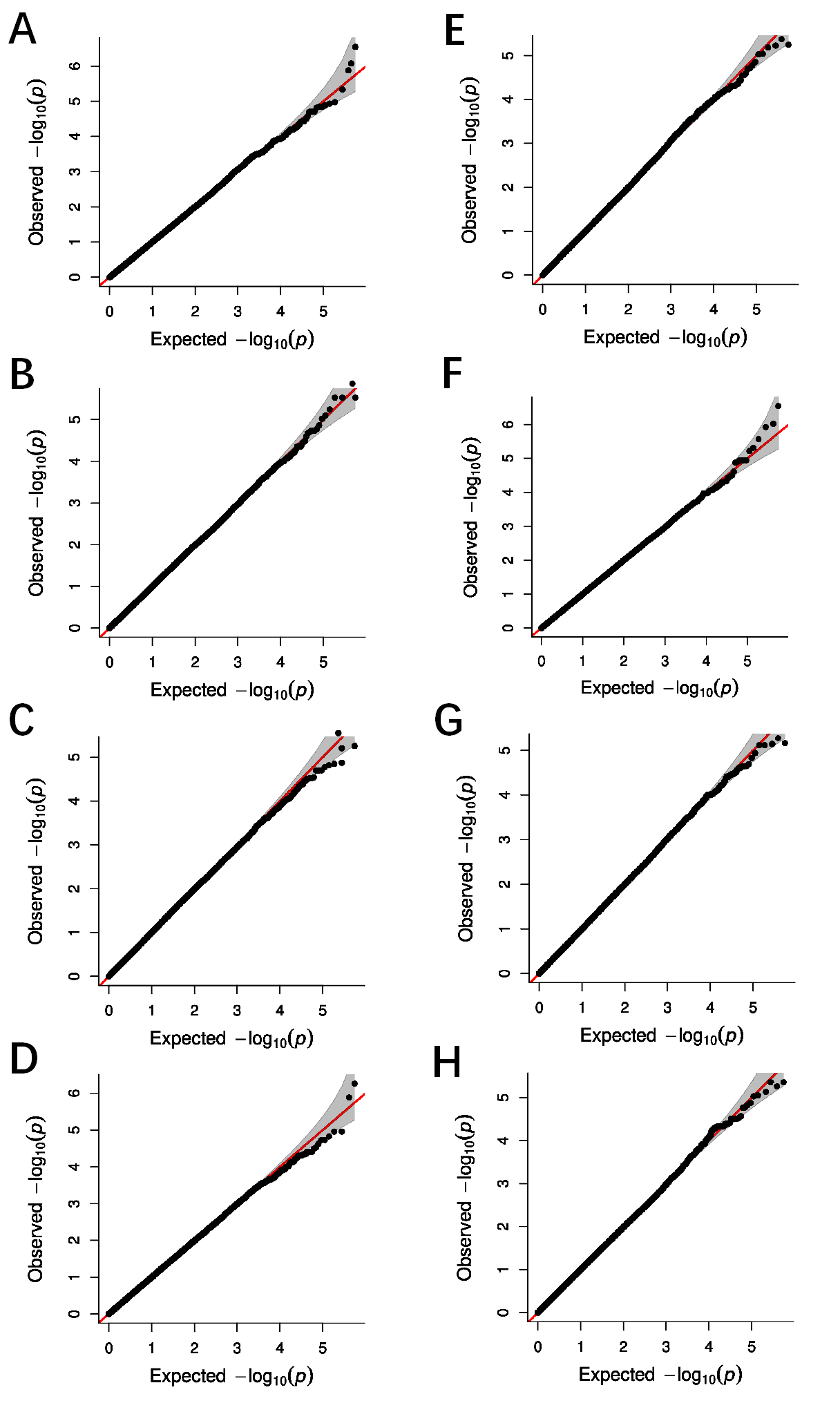

Supplement: Supplementary file 4 [file Image4.JPEG]

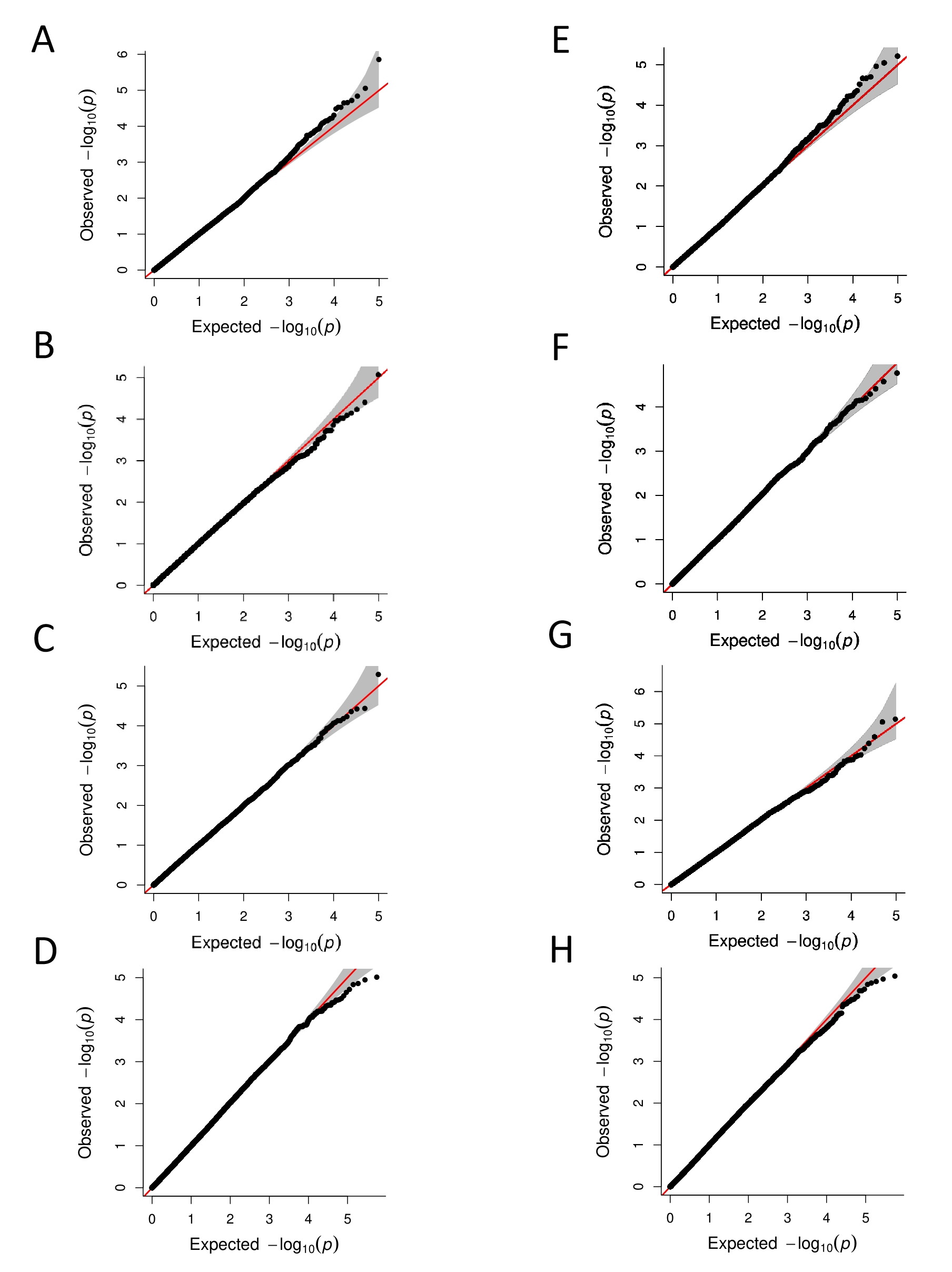

Supplement: Supplementary file 5 [file Image2.JPEG]
