# Supplementary material for: Genome-wide association study reveals the genetic basis of brace root angle and diameter in maize
Source: Front Genet. 2022 Oct 6;13:963852. doi: 10.3389/fgene.2022.963852 (PMC9582141; doi:10.3389/fgene.2022.963852)
Supplement: Supplementary file 2 [file Table3.doc]

**Table S3** SNPs chromosome positions and all candidate genes significantly correlated with BRA and BRD identified by MLM and BLINK.

| **Traits** | **SNP** | **Allele^a^** | **P-value** | **R^2^ (%)^b^** | **Gene** | **Arabidopsis homologous gene** | **Best annotations of Arabidopsis** | **Rice homologous gene** | **Best annotations of Arabidopsis** |
| --- | --- | --- | --- | --- | --- | --- | --- | --- | --- |
| BRA | chr3.S_218194187 | A/G | 4.91492E-07 | 2.40 | GRMZM2G156069 | AT5G53170.1 | (FTSH11) FTSH protease 11 | [LOC_Os01g43150.1](http://rice.plantbiology.msu.edu/cgi-bin/ORF_infopage.cgi?db=osa1r6&orf=LOC_Os01g43150.1) | OsFtsH9 FtsH protease homologue of AtFtsH11 expressed |
|  |  |  |  |  | GRMZM2G156126 | [AT3G61680.1](http://arabidopsis.org/servlets/TairObject?type=gene&name=AT3G61680.1) | alpha/beta-Hydrolases superfamily protein | [LOC_Os01g43140.1](http://rice.plantbiology.msu.edu/cgi-bin/ORF_infopage.cgi?db=osa1r6&orf=LOC_Os01g43140.1) | lipase putative expressed |
| BRA | chr10.S_4090245 | G/A | 5.36755E-07 | 1.91 | GRMZM2G088313 | Unknown | Unknown | Unknown | Unknown |
|  |  |  |  |  | GRMZM2G129775 | Unknown | Unknown | Unknown | Unknown |
|  |  |  |  |  | GRMZM2G129804 | [AT3G08740.1](http://arabidopsis.org/servlets/TairObject?type=gene&name=AT3G08740.1) | elongation factor P (EF-P) family protein | [LOC_Os12g02380.1](http://rice.plantbiology.msu.edu/cgi-bin/ORF_infopage.cgi?db=osa1r6&orf=LOC_Os12g02380.1) | elongation factor P putative expressed |
|  |  |  |  |  | GRMZM2G467576 | [AT3G10910.1](http://arabidopsis.org/servlets/TairObject?type=gene&name=AT3G10910.1) | RING/U-box superfamily protein | [LOC_Os11g02424.1](http://rice.plantbiology.msu.edu/cgi-bin/ORF_infopage.cgi?db=osa1r6&orf=LOC_Os11g02424.1) | LTPL9 - Protease inhibitor/seed storage/LTP family protein precursor expressed |
| 16-LN-BRA | chr8.S_107094508 | T/C | 2.99367E-07 | 3.50 | GRMZM2G040131 | [AT4G22745.1](http://arabidopsis.org/servlets/TairObject?type=gene&name=AT4G22745.1) | (MBD1) methyl-CPG-binding domain 1 | [LOC_Os05g33550.1](http://rice.plantbiology.msu.edu/cgi-bin/ORF_infopage.cgi?db=osa1r6&orf=LOC_Os05g33550.1) | methyl-binding domain protein MBD putative expressed |
| 16-JL-BRA | chr5.S_3504616 | C/T | 1.93558E-07 | 5.70 | GRMZM2G701288 | [AT3G04520.2](http://arabidopsis.org/servlets/TairObject?type=gene&name=AT3G04520.2) | (THA2) threonine aldolase 2 | [LOC_Os04g43650.1](http://rice.plantbiology.msu.edu/cgi-bin/ORF_infopage.cgi?db=osa1r6&orf=LOC_Os04g43650.1) | L-allo-threonine aldolase putative expressed |
|  |  |  |  |  | AC210013.4_FG003 | [AT5G13710.1](http://arabidopsis.org/servlets/TairObject?type=gene&name=AT5G13710.1) | (CPH, SMT1) sterol methyltransferase 1 | [LOC_Os03g59290.1](http://rice.plantbiology.msu.edu/cgi-bin/ORF_infopage.cgi?db=osa1r6&orf=LOC_Os03g59290.1) | C-methyltransferase putative expressed |
|  |  |  |  |  | GRMZM5G836683 | [AT5G13710.1](http://arabidopsis.org/servlets/TairObject?type=gene&name=AT5G13710.1) | (CPH, SMT1) sterol methyltransferase 1 | [LOC_Os03g59290.1](http://rice.plantbiology.msu.edu/cgi-bin/ORF_infopage.cgi?db=osa1r6&orf=LOC_Os03g59290.1) | C-methyltransferase putative expressed |
| 17-LN-BRA | chr6.S_112435160 | A/C | 5.45E-07 | 0.45 | GRMZM2G383680 | [AT5G39865.1](http://arabidopsis.org/servlets/TairObject?type=gene&name=AT5G39865.1) | Glutaredoxin family protein | [LOC_Os02g51370.1](http://rice.plantbiology.msu.edu/cgi-bin/ORF_infopage.cgi?db=osa1r6&orf=LOC_Os02g51370.1) | glutaredoxin putative expressed |
| BRD | chr9.S_140361053 | G/T | 4.24628E-07 | 7.70 | GRMZM2G179336 | [AT5G59020.1](http://arabidopsis.org/servlets/TairObject?type=gene&name=AT5G59020.1) | Protein of unknown function (DUF3527) | [LOC_Os03g16070.1](http://rice.plantbiology.msu.edu/cgi-bin/ORF_infopage.cgi?db=osa1r6&orf=LOC_Os03g16070.1) | expressed protein |
|  |  |  |  |  | GRMZM2G179329 | [AT5G05840.1](http://arabidopsis.org/servlets/TairObject?type=gene&name=AT5G05840.1) | Protein of unknown function (DUF620) | [LOC_Os03g16060.1](http://rice.plantbiology.msu.edu/cgi-bin/ORF_infopage.cgi?db=osa1r6&orf=LOC_Os03g16060.1) | expressed protein |
|  |  |  |  |  | GRMZM2G479243 | [AT1G31420.2](http://arabidopsis.org/servlets/TairObject?type=gene&name=AT1G31420.2) | (FEI1) Leucine-rich repeat protein kinase family protein | [LOC_Os03g16010.1](http://rice.plantbiology.msu.edu/cgi-bin/ORF_infopage.cgi?db=osa1r6&orf=LOC_Os03g16010.1) | BRASSINOSTEROID INSENSITIVE 1-associated receptor kinase 1 precursor putative expressed |
|  |  |  |  |  | GRMZM2G479260 | [AT1G53540.1](http://arabidopsis.org/servlets/TairObject?type=gene&name=AT1G53540.1) | HSP20-like chaperones superfamily protein | [LOC_Os03g15960.1](http://rice.plantbiology.msu.edu/cgi-bin/ORF_infopage.cgi?db=osa1r6&orf=LOC_Os03g15960.1) | hsp20/alpha crystallin family protein putative expressed |
| BRD | chr10.S_95437751 | A/C | 1.71503E-06 | 13.95 | GRMZM2G068350 | [AT1G27600.1](http://arabidopsis.org/servlets/TairObject?type=gene&name=AT1G27600.1) | (I9H, IRX9-L) Nucleotide-diphospho-sugar transferases superfamily protein | [LOC_Os04g01280.1](http://rice.plantbiology.msu.edu/cgi-bin/ORF_infopage.cgi?db=osa1r6&orf=LOC_Os04g01280.1) | glycosyltransferase family 43 protein putative expressed |
|  |  |  |  |  | GRMZM2G174736 | [AT4G24500.1](http://arabidopsis.org/servlets/TairObject?type=gene&name=AT4G24500.1) | hydroxyproline-rich glycoprotein family protein | [LOC_Os02g30640.1](http://rice.plantbiology.msu.edu/cgi-bin/ORF_infopage.cgi?db=osa1r6&orf=LOC_Os02g30640.1) | basic salivary proline-rich protein 2 precursor putative expressed |
| 16-LN-BRD | chr5.S_2275215 | G/A | 2.87E-07 | 2.40 | GRMZM2G143998 | [AT2G37900.1](http://arabidopsis.org/servlets/TairObject?type=gene&name=AT2G37900.1) | Major facilitator superfamily protein | [LOC_Os03g60850.1](http://rice.plantbiology.msu.edu/cgi-bin/ORF_infopage.cgi?db=osa1r6&orf=LOC_Os03g60850.1) | peptide transporter PTR2 putative expressed |
|  |  |  |  |  | GRMZM2G144008 | [AT1G07410.1](http://arabidopsis.org/servlets/TairObject?type=gene&name=AT1G07410.1) | (ATRAB-A2B, ATRABA2B, RAB-A2B, RABA2b) RAB GTPase homolog A2B | [LOC_Os03g60870.1](http://rice.plantbiology.msu.edu/cgi-bin/ORF_infopage.cgi?db=osa1r6&orf=LOC_Os03g60870.1) | ras-related protein putative expressed |
|  |  |  |  |  | GRMZM2G144020 | [AT3G09350.1](http://arabidopsis.org/servlets/TairObject?type=gene&name=AT3G09350.1) | (Fes1A) Fes1A | [LOC_Os03g60780.1](http://rice.plantbiology.msu.edu/cgi-bin/ORF_infopage.cgi?db=osa1r6&orf=LOC_Os03g60780.1) | armadillo/beta-catenin-like repeat containing protein expressed |
|  |  |  |  |  | GRMZM2G445169 | [AT2G39700.1](http://arabidopsis.org/servlets/TairObject?type=gene&name=AT2G39700.1) | (ATEXP4, ATEXPA4, ATHEXP ALPHA 1.6, EXPA4) expansin A4 | [LOC_Os03g60720.1](http://rice.plantbiology.msu.edu/cgi-bin/ORF_infopage.cgi?db=osa1r6&orf=LOC_Os03g60720.1) | expansin precursor putative expressed |
|  |  |  |  |  | GRMZM2G144042 | [AT1G07570.1](http://arabidopsis.org/servlets/TairObject?type=gene&name=AT1G07570.1) | (APK1, APK1A) Protein kinase superfamily protein | [LOC_Os03g60710.1](http://rice.plantbiology.msu.edu/cgi-bin/ORF_infopage.cgi?db=osa1r6&orf=LOC_Os03g60710.1) | protein kinase domain containing protein expressed |
|  |  |  |  |  | GRMZM2G144071 | [AT4G21065.1](http://arabidopsis.org/servlets/TairObject?type=gene&name=AT4G21065.1) | Tetratricopeptide repeat (TPR)-like superfamily protein | [LOC_Os03g60690.1](http://rice.plantbiology.msu.edu/cgi-bin/ORF_infopage.cgi?db=osa1r6&orf=LOC_Os03g60690.1) | pentatricopeptide putative expressed |
| 16-JL-BRD | chr4.S_237033434 | G/T | 1.86264E-07 | 4.90 | GRMZM2G012262 | [AT1G71260.1](http://arabidopsis.org/servlets/TairObject?type=gene&name=AT1G71260.1) | (ATWHY2, WHY2) WHIRLY 2 | [LOC_Os02g06370.1](http://rice.plantbiology.msu.edu/cgi-bin/ORF_infopage.cgi?db=osa1r6&orf=LOC_Os02g06370.1) | whirly transcription factor domain containing protein expressed |
|  |  |  |  |  | GRMZM2G012143 | [AT3G54690.1](http://arabidopsis.org/servlets/TairObject?type=gene&name=AT3G54690.1) | Sugar isomerase (SIS) family protein | [LOC_Os02g06360.1](http://rice.plantbiology.msu.edu/cgi-bin/ORF_infopage.cgi?db=osa1r6&orf=LOC_Os02g06360.1) | uncharacterized protein CPn_0526/CP_0226/CPj0526/CpB0547 putative expressed |
| 16-JL-BRD | chr5.S_180407216 | G/C | 2.29814E-07 | 8.35 | GRMZM2G038073 | [AT1G25580.1](http://arabidopsis.org/servlets/TairObject?type=gene&name=AT1G25580.1) | (ANAC008, SOG1) NAC (No Apical Meristem) domain transcriptional regulator superfamily protein | [LOC_Os02g38130.1](http://rice.plantbiology.msu.edu/cgi-bin/ORF_infopage.cgi?db=osa1r6&orf=LOC_Os02g38130.1) | no apical meristem protein putative expressed |
|  |  |  |  |  | GRMZM2G079682 | [AT3G27540.1](http://arabidopsis.org/servlets/TairObject?type=gene&name=AT3G27540.1) | beta-1 4-N-acetylglucosaminyltransferase family protein | [LOC_Os02g38140.1](http://rice.plantbiology.msu.edu/cgi-bin/ORF_infopage.cgi?db=osa1r6&orf=LOC_Os02g38140.1) | glycosyl transferase family 17 protein putative expressed |

^a^ Major/minor alleles, underlined bases indicate favorable alleles.

^b^ Percentage of phenotypic variation explained by the cumulative effect of a single significant SNP.
