# Supplementary material for: Genome-wide association study reveals the genetic basis of brace root angle and diameter in maize
Source: Front Genet. 2022 Oct 6;13:963852. doi: 10.3389/fgene.2022.963852 (PMC9582141; doi:10.3389/fgene.2022.963852)
Supplement: Supplementary file 6 [file Table2.docx]

**Table S2** Phenotypic variation distribution, analysis of variance, and broad-sense heritability of BRA and BRD in three individual environments.

| **Traits^a^** | **Env^b^** | **Means ± SD** | **Range^c^** | **Variance component^d,e^** | | | ***h*^2f^** |
| --- | --- | --- | --- | --- | --- | --- | --- |
|  |  |  |  | **Genotype(G)** | **Environment (E)** | **G×E** |  |
| BRA | 16LN | 56.54±16.21 | 20.85-82.67 | 48.96^*^ | 14.27^*^ | 20.82^*^ | 0.82 |
|  | 16JL | 51.42±18.11 | 20.88-85.97 | 27.35^*^ | 11.65^*^ | 29.71^*^ | 0.64 |
|  | 17LN | 47.69±15.61 | 16.88-84.10 | 52.82^*^ | 16.34^*^ | 23.28^*^ | 0.76 |
| BRD | 16LN | 0.46±0.08 | 0.23-0.65 | 29.85^*^ | 18.37^*^ | 11.29^*^ | 0.64 |
|  | 16JL | 0.43±0.09 | 0.20-0.65 | 17.94^*^ | 19.56^*^ | 14.52^*^ | 0.36 |
|  | 17LN | 0.46±0.08 | 0.30-0.66 | 28.56^*^ | 15.95^*^ | 12.85^*^ | 0.47 |

^a^ *BRA* brace root angle, *BRD* brace root diameter.

^b^ *16LN* Liaoning Province in 2016, *16JL* Jilin Province in 2016, *17LN* Liaoning Province in 2017.

^c^ The range of BRA and BRD in three individual environments.

^d^ *G* and *E* indicate genotype and environment, respectively, and *G× E* indicate interaction of G and E.

^e^ ^*^ represents significant difference at the 0.01 level.

^f^ Family mean-based broad-sense heritability.
